# The Relationship between Short Video Flow, Addiction, Serendipity, and Achievement Motivation among Chinese Vocational School Students: The Post-Epidemic Era Context

**DOI:** 10.3390/healthcare11040462

**Published:** 2023-02-05

**Authors:** Weiguaju Nong, Zhen He, Jian-Hong Ye, Yu-Feng Wu, Yu-Tai Wu, Jhen-Ni Ye, Yu Sun

**Affiliations:** 1 School of Education, Guangxi University of Foreign Languages, Nanning 530222, China; 2 Faculty of Education, Beijing Normal University, Beijing 100875, China; 3 National Institute of Vocational Education, Beijing Normal University, Beijing 100875, China; 4 Office of Physical Education, Ming Chi University of Technology, New Taipei City 243303, Taiwan; 5 Office of Physical Education, Soochow University, Taipei City 111002, Taiwan; 6 Graduate Institute of Technological & Vocational Education, National Taipei University of Technology, Taipei City 106344, Taiwan; 7 Department of Industrial Education, National Taiwan Normal University, Taipei City 106, Taiwan

**Keywords:** achievement motivation, interaction of person-affect-cognition-execution (I-PACE), relying on short video APP, serendipity (小确幸), short video addiction, short video flow, short video problematic use, vocational school

## Abstract

Since the COVID-19 outbreak, people have been spending more time in the online world because of restrictions on face-to-face communication due to epidemic prevention controls. This has also brought the issue of Internet addiction, including the overuse and negative effects of short videos, to the forefront of attention. Past research has found that Internet addiction has a negative impact on well-being. However, there is a special concept of positive emotion called “serendipity” (小确幸). Serendipity provides a small, fleeting but positive experience, yet it is often associated with negative perceptions from an outside perspective. However, the relationship between short video addiction and serendipity is not yet known. Based on this, a theoretical model was developed in the context of the I-PACE model. To understand the relationship between short video addiction and serendipity among college students, in this study, we conducted snowball sampling and distributed online questionnaires using the Wenjuanxing platform. The target population of the questionnaire distribution was vocational college students in China, of whom 985 valid study participants responded, yielding a valid return rate of 82.1%. Of the respondents, 410 (41.6%) were male and 575 (58.4%) were female. The results were as follows: a. short video flow had a positive relationship with serendipity, a negative relationship with achievement motivation, and a positive effect on short video addiction; b. short video addiction had a positive effect on serendipity and a negative effect on achievement motivation; and c. serendipity had a negative impact on achievement motivation. This shows that short video addiction, like other Internet addictions, can have a negative impact on students’ learning.

## 1. Introduction

The COVID-19 pandemic has significantly disrupted the daily lives of people around the world, and isolation measures within the home have increased the use of digital entertainment [1]. The Chinese government has also developed and implemented a series of effective outbreak prevention and control strategies (e.g., studying at home, mandating less physical social interaction, and the suspension of restaurant operations) to contain the spread of the virus since the outbreak [2], which have resulted in even more time spent online.

Due to the COVID-19 pandemic, infection control measures have allowed young people to spend more time at home and make extensive use of Internet technologies. Moreover, the information resources available on the Internet today may meet the needs of adolescents who enjoy receiving and sharing personal and social information and new knowledge with others [3]. Therefore, many social media or online social platforms, including short videos, have become a place for knowledge sharing or learning. However, in addition, as people may rely heavily on digital platforms for socialization, entertainment, and information sharing, COVID-19 may have changed their digital lifestyles [4]. The use of social media by adolescents has been increasing and has various potential effects on their development [5]. Specifically, because people spend more time using the Internet, this also leads to associated adverse psychological and behavioral effects [6]. Therefore, while the benefits of the Internet are widely recognized, the negative effects of uncontrolled Internet use have been a concern for researchers [7]. “Uncontrolled” also means that student users cannot watch a video within the expected and planned usage time, resulting in the disruption of their daily routine. Research has also shown that maladaptive patterns of Internet use constitute behavioral addiction [8]. Therefore, Internet addiction can become a serious problem for adolescents [1]. In addition, excessive use of the Internet has been reported to have a negative impact on young people’s academic performance, family relationships, and emotional development [9].

Smartphone addiction has been a major problem for children and adolescents for the past few decades, and it may have been exacerbated by the COVID-19 outbreak, thus posing a threat to this demographic’s physical and mental health [10] and intensifying different related behavioral addictions. Internet addiction has attracted attention from the mass media and researchers, and this attention has paralleled the growth of computer (Internet) access [11]. For example, with the explosion of Chinese video applications (apps) such as Jitterbug and Racer, short video applications have swept across schools throughout the world. However, the immersive nature of such software predisposes students to problematic use and even addiction [12]. Due to the variety of short videos and their corresponding involvement norm, the rate of such videos’ consumption is growing, and students’ addiction to short videos is also causing many possible problems in terms of learning outcomes [13]. Therefore, as the use of short video media is very common in our daily life, people are becoming increasingly worried about the negative effects of its consumption in terms of, for instance, spending too much time on short video media.

As such, the behavioral problems triggered during the COVID-19 pandemic may have had a very undesirable influence on the mental health of some individuals [14]. At the same time, issues related to mental health and digital lifestyle may become a major public health problem [4,15], so it is necessary to pay attention to the mental health problems caused by people’s use of digital technology products in the context of the epidemic. Although we have moved from the pandemic to a post-epidemic era, China has taken a variety of preventive measures in response to geographic outbreaks during this unstable period. Therefore, this study was conducted in the context of the post-epidemic era.

In recent years, there have been many novel theoretical frameworks that have helped explain the usage behavior of Internet tools/systems. Among them, the Interaction of Person–Affect–Cognition–Execution (I-PACE) is a comprehensive model that attempts to explain how people use and overuse the Internet [16] with respect to, for example, online gaming, online gambling, online shopping, online communication, and the use of specific Internet programs [17]. According to Brand et al., who developed the I-PACE model, addiction is described as people’s emotional and cognitive reactions to circumstances (i.e., internal or external stimuli), involving cognitive biases, coping styles, attentional biases, and cravings [18]. These factors affect their emotional and cognitive responses to situational triggers, leading to deterioration in executive functioning [19]. In addition, Internet addiction can mediate the relationship between personal characteristics and problematic technology use, prompting this response to evolve into executive functioning, that is, a reduction in executive functioning and inhibitory control that may lead to excessive Internet use [20]. Later in the addiction’s development, although this change may be ongoing, the above responses may become progressively strong, leading to habitual actions that may feel involuntary in some cases [21]. Therefore, this study used the I-PACE model as a theoretical framework to explore the causes of short video addiction and the influence of watching short videos on students.

The convenience, entertainment, and attractiveness of short video apps lead people to easily immerse themselves in the apps and enjoy them, and they then experience a state of flow [13]. A related consequence of flow is that users lose their sense of time [22]. This may lead to the development of short video addiction, which refer to individuals’ strong or uncontrollable fixation on videos available on short video apps. Short video addiction is defined as an addictive behavior in which users engage in the use of short video software dependently, inappropriately, or excessively [13]. Addictive behaviors are thought to be associated with cue responses and craving [21], which are compulsive, seeking behaviors. Such seeking behaviors can invoke the desire to replace other important activities with the addictive one [23]. As a result, addictive behaviors have unwanted and negative consequences for users [10].

In addition, in the world of short videos, besides bringing users an inextricable sense of immersion, watching videos and interacting with live broadcasters simultaneously also gives users a rich sense of satisfaction; this sense of satisfaction and little things in life is known as serendipity (小确幸). The topic of serendipity has been spreading rapidly since 2012 in the Chinese-speaking regions of China, Taiwan, and Hong Kong, and seems to have gradually penetrated the mindset of the general public [24]. Serendipity was mentioned in an essay in “Afternoon on Langerhans Island”, written by Japanese author Haruki Murakami, in which it denoted “a small but definite happy thing in life” [25]. The feeling of serendipity lies in the little things in life, and each experience of serendipity lasts from 3 s to 3 min [26]. In addition, it can also be a small blessing to include time and factors to escape and temporarily indulge in smaller objects or things [27]. From the above, serendipity can be conceptualized as a kind of small fortune and happiness in life, and also refers to the fleeting beauty. Therefore, since the introduction of the term serendipity, it has been sought after by netizens. The term serendipity has thus become common in our lives in recent years. In these recessionary times, due to anxiety about the future, the pursuit of “big happiness” seems to be out of reach and so people turn to serendipity to gain satisfaction [28]. Moreover, it is generally accepted in modern society that many young people nowadays are pursuing serendipity [29]. Some research has indicated that students’ well-being has a positive influence on accommodating new information, confronting new challenges, and sustaining learning as a motivation for sustainability [30]. However, the role of serendipity is still unknown. Therefore, in this study, we investigated the effects of short video flow and short video addiction on the effects of serendipity and, in turn, the effects of serendipity on students’ learning motivation.

Students’ motivation to learn has long been a major focus of educational psychology theory and research [31]. Motivation inspires and guides action and is, therefore, closely associated with many important developmental outcomes [32]. For example, according to the achievement motivation theory proposed by Atkinson, people with high achievement motivation care about success, tend to engage in achievement-related activities, and prefer moderately difficult tasks [33]. Therefore, students’ motivation to enjoy and continue learning has been identified as a key factor of academic success [13]. However, experts believe that as students’ physical interactions with classmates and teachers have reduced, their achievement motivation may also have worsened since the outbreak of the COVID-19 pandemic [34]. In addition, some studies have found that smartphone addiction [35] and online game addiction have a negative impact on students’ motivation to learn [36]. Therefore, in this study, achievement motivation was considered as an important outcome variable.

In summary, the purpose of this research was to identify the association between short video flow, addiction, serendipity, and achievement motivation of vocational students in the post-epidemic era through the theoretical framework of the I-PACE model.

## 2. Theoretical Foundations, Research Hypotheses, and Models

### 2.1. I-PACE Model

The I-PACE model is a process model according to which susceptibility variables, cognitive and affective responses to external or internal stimuli, and executive and inhibitory control lead to people’s decision-making behavior using certain web applications and consequential choices [18]. Therefore, this model has been used to describe, develop, and maintain the neurobiological and psychological processes in the context of the addictive use of particular Internet applications [21]. Furthermore, according to the arguments of the I-PACE model, Internet-addictive behavior occurs in the interaction between variables associated with an individual’s tendency and their opinion of a particular situation, while the triggering variables represent the core characteristics of the individual, and the range of cognitive and emotional responses elicited by these susceptibility features or external Internet-associated stimuli can affect the extreme Internet use behaviors associated with such responses [21,37,38]. Moreover, since media use is one of the many activities on the Internet, the I-PACE model can be used to investigate problematic behaviors related to media use [20]. Thus, this study used the I-PACE model as a theoretical framework for model construction.

### 2.2. Research Model

**Figure 1:**
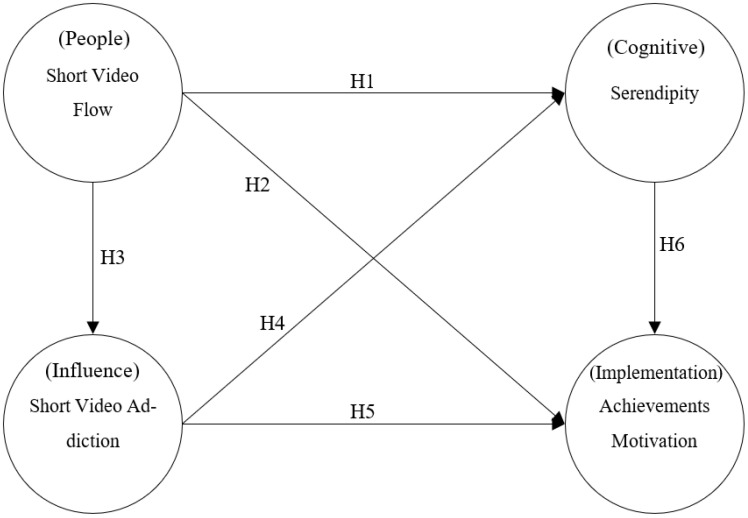
Research Model.

Internet usage barriers involve person–influence–cognition–execution interactions [18]. More specifically, in the I-PACE model, personal feelings affect people’s state and cognitive reflections, and eventually their thoughts on action change as well. Therefore, the variables used in the I-PACE model will be different for different research topics. According to the above concepts, this research proposes six hypothetical paths under the framework of the I-PACE model to construct an affective–behavioral–outcome model to explore the association between short video flow, short video addiction, serendipity, and achievement motivation, as indicated in Figure 1.

### 2.3. Research Hypotheses

#### 2.3.1. The Relationship between Short Video Flow and Serendipity

According to the flow theory, when people undergo a highly emotionally positive experience, they can be described as experiencing “flow”, where flow is the optimal state of being in which people are so absorbed in and dedicated to an event that they lose their awareness of time and self-awareness, enjoying each minute and being guided to repeat the feeling [39]. Thus, flow is seen as a psychological state (immersive enjoyment) that generates a psychological energy and enhances motivation to achieve the best conditions for experiencing flow again [22]. Across all of the different groups studied, the respondents reported a very similar subjective experience that was enjoyed to such a degree that they were willing to go out of their way to experience it again [40]. Flow is the main source or component of happiness [41]. Flow-based activities have been shown to promote long-term well-being to a greater extent than low-involvement, passive activities [42]. In addition, there is a growing body of research linking flow experiences to well-being [43]. According to the above findings, this study used short video flow to explore the participants’ relationship with serendipity according to the following hypothesis:

 **Hypothesis 1 (H1).**  *Short video flow is positively related to serendipity.*

#### 2.3.2. The Relationship between Short Video Flow and Achievement Motivation

Achievement motivation is defined as a person’s desire to achieve goals based on a set of criteria, because motivation is a key to academic success, which may be influenced by intrinsic phenomena, situations, goals, and other factors [44]. Whereas flow is a positive or negative motivational effect on the performance of an activity; when motivation is positive, individuals may engage in an activity simply for enjoyment [45]. Whenever students are watching short videos, they experience a flow that makes them want to watch said videos more consistently. Thus, in such an optimal experience, students enter a psychological state in which they are so highly engaged in task-driven activities that nothing else seems to matter [39]. That is to say, when students experience short video flow, they will probably neglect their studies. In contrast, previous research has found that short video flow has an indirect negative effect on learning motivation [13]. According to the above findings, this study used short video flow to explore the relationship between participants’ motivation in achievement in accordance with the following hypothesis.

 **Hypothesis 2 (H2).**  *Short video flow is negatively related to achievement motivation.*

#### 2.3.3. The Relationship between Short Video Flow and Short Video Addiction

Social media allows users to interact with and access various types of information online alongside people who may be acquaintances or strangers, which leads to prolonged periods spent online [5]. In addition, because the effects of the pandemic have lasted for an extensive period, the use of television and electronics continues to be prolonged, which may lead to behavioral addiction [46]. The mechanism behind this influence can be explained by flow theory. The nature of the flow state is self-reinforcing, as individuals experience the feeling of happiness, satisfaction, and pleasure, and are thus motivated to repeat the activity that invoked such feelings [22]. Furthermore, according to the I-PACE model, gratification leads to the active reinforcement of dysfunctional coping styles (Brand et al., 2016) [18] such that people’s emotional and cognitive responses lead to decisions causing them to act in a particular way [21]. That is, when users experience a high level of enjoyment when watching short videos, their desire to watch such videos will continue to increase with each viewing. Based on the above findings, this study explored the relationship between participants’ addiction to short videos and short video flow in accordance with the following hypothesis:

 **Hypothesis 3 (H3).**  *Short video flow is positively related to short video addiction*.

#### 2.3.4. The Relationship between Short Video Addiction and Serendipity

While it has been suggested that watching videos (passive use) decreases life satisfaction and reduces positive effects, online posting (active use) increases life satisfaction [47]. However, during the pandemic, people spent a great deal of time watching television and their electronic devices because the source of pleasure was limited to indoor activities [46]. Moreover, when people develop Internet addiction, it creates a state wherein they feel they need the Internet to function in their daily lives [48]. Historically, the dominant view of happiness or the good life has viewed the achievement of such states as being attainable through maximizing pleasure and minimizing pain [49]. Therefore, when people think that the meagre content of a short video brings them happiness, it will also likely prompt them to seek serendipity. In addition, addiction experts have observed that when associating emotional states with addictive activities, addicts tend to confuse happiness with well-being [50]. According to the above findings, this study explored the relationship between short video addiction and participants experiencing serendipity in accordance with the following hypothesis:

 **Hypothesis 4 (H4).**  *Short video addiction is positively related to serendipity*.

#### 2.3.5. The Relationship between Short Video Addiction and Achievement Motivation

Motivation is linked to belief systems with different activation patterns depending on the context and personal factors [51], and, in addition to being activated, such patterns may also appear to inhibit motivation. Related research on Internet addiction has reported negative effects of Internet addiction on academic motivation [52]. Online game addiction is negatively associated with students’ motivation to learn [36]. Addicts choose to forfeit their daily behaviors and devote their time to their activities on the Internet [53]. Therefore, addictive games may interfere with the way people learn, and players may neglect their homework [54]. Some research has also discovered that short video addiction negatively affects learning motivation [13]. Although there are still few empirical studies on short-form video addiction, according to the above findings, this study on short video addiction explored the relationship between participants’ addiction and achievement motivation in accordance with the following hypothesis:

 **Hypothesis 5 (H5).**  *Short video addiction is negatively related to achievement motivation*.

#### 2.3.6. The Relationship between Serendipity and Achievement Motivation

Happiness plays an important role in the academic environment [55]. It is widely believed that happiness can provide a great motivation for someone to achieve higher performance [56]. Students’ happiness has an affirmative influence on acquiring new knowledge, encountering new challenges, and sustaining their motivation to learn [57]. In addition, a study also found a direct and noteworthy association between happiness and achievement motivation among university students [58]. However, the key to achievement motivation is the desire to perform the activities in question well or better than others [59]. However, no matter how the idea of serendipity is applied to any given scenario, the characteristics of “easy”, “easy to achieve”, and “easy to satisfy” are, in fact, not separated from said concept. Explained from the perspective of the theory of basic psychological needs, individuals may feel that their basic psychological needs (i.e., autonomy, competence, and interpersonal relationships) are met and, therefore, may be less motivated to pursue higher achievement performance [60]. As a result, students may not focus on achieving their academic goals when they are chasing serendipity or focusing on the small and beautiful things. Based on the above findings, this study examined the relationship between participants’ motivation for achievement and serendipity, in accordance with the following hypothesis:

 **Hypothesis 6 (H6).** *Serendipity is negatively related to achievement motivation*.

## 3. Methodology

### 3.1. Research Procedure

This study used snowball sampling to distribute the questionnaire online via Wenjuanxing (online questionnaire platform). The questionnaire was distributed to students in vocational colleges in China, and respondents were asked to forward the link to their peers who also regularly watched short videos. Questionnaires were collected from 20 February 2022, until the web link was closed after 1200 questionnaires were collected. On the first page of the electronic questionnaire, we provided informed consent instructions detailing the purpose and meaning of the survey, and that the data collected would only be used within the scope of the study and would be anonymous. Participants were also asked not to submit any personally identifiable information on the questionnaire. Participants were considered to have given their electronic informed consent when they agreed to participate in the study and then clicked on the next page to answer the questions. Therefore, the participants participated in this study of their own free will. In addition, the email addresses of the corresponding authors were published on the first page of the questionnaire so that the participants could consult them at any time.

### 3.2. Measurement

This study was a quantitative validation study; data were collected by questionnaire developed from past theories and research. The questionnaire was reviewed by three educational experts. The content of the questionnaire was analyzed with a 5-point Likert scale (assessed from 1 to 5 for strongly disagree to strongly agree). The higher the score, the greater the perception of the content of the variable.

#### 3.2.1. Short Video Flow

In this study, short video flow is regarded as the state of high enjoyment and engagement that students experience when watching short videos. Based on this meaning, this study referred to and adapted the Flow Scale from Hong et al. [61] with eight items to measure individuals’ perception of having highly engaging and pleasurable experiences while watching short videos. Examples of the questions are as follows: “When I watch a short video, I will keep watching it in order to forget myself” and “After watching a short video, I find that I can’t watch it again and I feel a sense of loss”.

#### 3.2.2. Short Video Addiction

In this study, short video addiction refers to the situation wherein students are unable to control themselves with respect to watching short videos appropriately. Based on this, this study referred to and adapted the Game Addiction Scale from Ye et al. [13], incorporating eight questions to analyze the participants’ perceptual levels of self-viewing short videos in order to determine the degree of addiction. Examples of the questions are as follows: “I will drop what I need to finish or do and spend my time watching short videos” and “I will sacrifice my sleep at night because of watching short videos”.

#### 3.2.3. Serendipity

In this study, serendipity is defined as the short-term but positive feelings that students experience after watching short videos. Based on this, this study adapted a very short version of Luo Lu’s Chinese Well-Being Scale [62] with eight questions to measure participants’ perceptions of satisfaction after watching short videos. Examples of the questions are: “Watching short videos has made my life a good experience” and “Watching short videos has enabled me to enjoy my current state of life more”.

#### 3.2.4. Achievement Motivation

In this study, achievement motivation refers to students’ perspectives on the pursuit of higher academic goals. In accordance with this definition, this study referred to and adapted the Achievement Motivation Scale from Ye et al. [63], using nine items to measure individuals’ perceived levels of motivation to achieve higher learning goals. Examples of the questions are: “I am not satisfied with my current learning performance or achievements and will seek higher challenges whenever possible” vs. “I am eager to learn and willing to spend time to increase my knowledge absorption”.

## 4. Results

IBM’s SPSS 23.0 and AMOS 20.0 programs were used to analyze the questionnaire data, to reanalyze the reliability and validity of the scales used in the study, and to conduct a validated factor analysis.

### 4.1. Participants

The number of participants included in this study is 1200. After 215 invalid data were deleted, the number of valid study participants was 985, with a valid return rate of 82.1%. Of the participants, 410 (41.6%) were male and 575 (58.4%) were female; 251 (25.5%) were vocational college students and 734 (74.5%) were attempting to earn vocational bachelor’s degrees; and 217 students (22%) were in first grade, 435 (44.2%) were in second grade, 228 (23.1%) were in third grade, and 105 (10.7%) were in fourth grade. Regarding weekly viewing, 95 (9.6%) reported watching short videos 1–3 days per week, 219 (22.2%) watched short videos 4–6 days per week, and 671 (68.1%) were daily viewers. Regarding average daily viewing time, 69 (6.8%) spent less than 1 h, 580 (58.9%) spent 1–3 h, 201 (20.4%) spent 3–5 h, and 137 (13.9%) spent more than 5 h. The mean age of the participants was 20.42 years (standard deviation of 1.92 years).

### 4.2. Item Analysis

Before SEM, it is necessary to verify that the fit of each measurement model meets acceptable standards. In this study, item analysis was used to examine the measurement model, and the index values were assessed as follows: χ2/*df* (Chi-Square / degrees of freedom) values should be less than 5; RMSEA (root-mean-square error of approximation) has to be lower than 0.10; GFI (goodness of fit index) and AGFI (adjusted goodness of fit index) have to be greater than 0.80; and questions with factor loadings (FL) greater than 0.50 should be removed from the original questionnaire [64,65]. As shown in Table 1, the deletion results were as follows: short video flow was reduced from eight to five questions; short video addiction from eight to five questions; serendipity from seven to five questions; and achievement motivation from seven to five questions. External validity measures of the questions in this study were used to discriminate the explanatory range of the study [66], and the values of all respondents for each question were divided into the bottom 27% and top 27% for a *t* test; the external validity is significant when the *t*-value is greater than 3 (*** p < 0.001). Table 1 shows that the *t*-values for the constructs ranged from 24.40 to 36.76 (*** p < 0.001), which signifies that all the retained items achieved external validity [67].

### 4.3. Construct Reliability and Validity Analysis

Cronbach’s α was used in this study to verify the reliability, and the internal consistency was measured with composite reliability (CR). According to Hair et al., the acceptable value for Cronbach’s α and CR should be higher than 0.70 to be considered reliable [64]. The Cronbach’s α and CR values for this study were between 0.81 to 0.92, which met the recommended criteria, as shown in Table 2.

The factor loading (FL) and average variance extracted (AVE) were used to measure convergent validity. Hair et al. specified that the FL value must be greater than 0.50, and if it is smaller than this value, the items should be deleted, while all the items that meet the recommended criteria can be retained [64]. In this study, the FL values were between 0.68 and 0.81 (see Table 2). Hair et al. recommended that the value of the AVE should be larger than 0.50 so that the construct’s convergent validity is met [68]. The AVE values for this study were between 0.50 to 0.70, as shown in Table 2.

Awang indicated that the AVE root number value of each construct must be larger than the Pearson correlation coefficient value of other constructs, which indicates that the construct has discriminant construct validity [69]. The following results indicate that every construct in this study had discriminant validity, as shown in Table 3.

### 4.4. Model Fit Analysis

Before performing model validation, we confirmed whether the model had an acceptable fit, wherein the fit indicators are as follows: χ^2^/*df* must be smaller than 5 [64]; RMSEA must be smaller than 0.1; GFI, AGFI, NFI, NNFI, CFI, IFI, and RFI must be larger than 0.80 [70]; and the PNFI and PGFI equivalents must be larger than 0.50 [64]. The fit index values in this study were as follows: χ^2^ = 503.9, *df* = 164, χ^2^/*df* = 3.07, RMSEA = 0.05, GFI = 0.95, AGFI = 0.94, NFI (normed fit index) = 0.95, NNFI (non-normed fit index) = 0.95, CFI (comparative fit index) = 0.97, IFI = 0.97, RFI = 0.95, PNFI (parsimonious normed fit index) = 0.82, and PGFI (parsimony goodness of fit index) = 0.74, and the results of the analysis indicated that the fit values of the hypothetical model proposed in this study were at an acceptable level.

### 4.5. Model Validation Analysis

**Figure 2:**
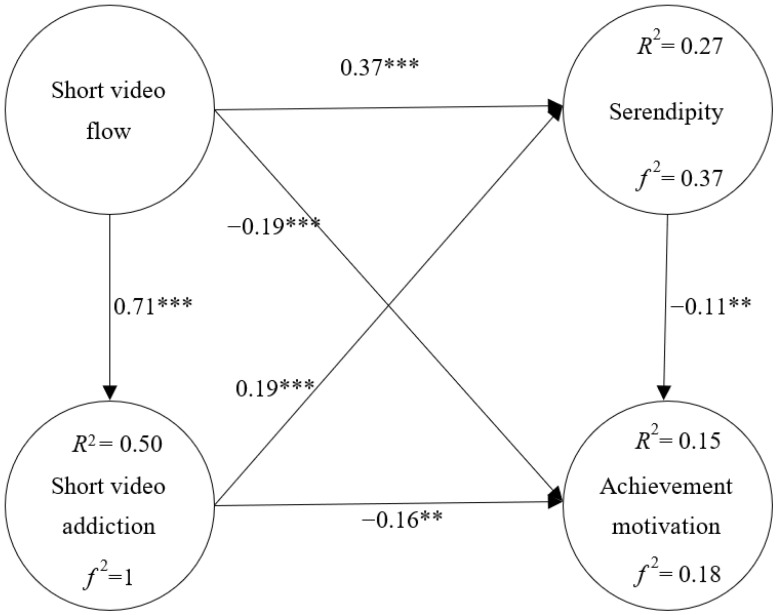
Model Validation. ** *p* < 0.01, *** *p* < 0.001.

This study analyzes the relationship of variables in the proposed conceptual model through path analysis, and then examines the model. The results of the study showed that short video flow was positively related to serendipity (β = 0.37 ***), negatively related to achievement motivation (β = −0.19 ***), and positively affected by short video addiction (β = 0.71 ***). Short video addiction was positively related to serendipity (β = 0.19 ***) and negatively related to achievement motivation (β = −0.16 **). Serendipity had a negative effect on achievement motivation (β = −0.11 **), as shown in Figure 2.

The explanatory power of short video flow with respect to short video addiction is 50% and f2 is 1. The explanatory power of short video flow and short video addiction with respect to serendipity is 27% and *f*^2^ is 0.37. The explanatory power of short video flow, short video addiction, and serendipity with respect to achievement motivation is 15% and *f*^2^ is 0.18, as shown in Figure 2.

## 5. Discussion

### 5.1. Short Video Flow Was Positively Related to Serendipity

The results of this study showed that short video flow was positively related to serendipity. This also means that H1 is supported. That is, when students experience a high level of flow when watching short videos, they will experience greater serendipity. This is because serendipity stems from people experiencing happy things themselves, and much of the content of short videos has an entertaining effect. This result is also in line with flow theory [39], which states that when people enter a highly emotionally positive flow experience, they are so absorbed and focused on an activity that they enjoy every minute of it and are compelled to try to repeat the feeling. This is also in line with the core values that serendipity brings to people. In addition, Csikszentmihalyi stated that flow generates a psychological energy that strengthens one’s motivation to experience this enjoyment again [22]. Thus, Schiffer and Roberts suggested that engaging in flow-based activities can promote long-term well-being [42]. In addition, Mao et al. also indicated that there is a growing body of research confirming that the flow experience is related to well-being [43]. In short, the positive relationship between short video flow and serendipity is supported by both research analysis and the literature.

### 5.2. Short Video Flow Was Negatively Related to Achievement Motivation

In this study, the results indicated that short video flow was negatively related to achievement motivation. This also means that H2 is supported. That is, when students experienced high levels of flow when watching short videos, they were less motivated to achieve. Therefore, as it is not a learning task, the flow experience of the short video will not increase students’ motivation to study harder. Such a result is also in line with Rheinberg and Engeser’s suggestion that flow has a positive or negative motivational effect on the performance of an activity, and when the motivation is positive, an individual may engage in an activity simply for enjoyment [45]. Thus, with such an optimal experience, students enter a psychological state in which they are so highly engaged in task-driven activities that nothing else seems to matter [39]. Therefore, when students watch short videos, they experience a flow that makes them want to watch them more consistently, resulting in a lack of motivation to learn. This coincides with Ye et al.’s finding of an indirect negative effect of short video flow on learning motivation [13]. In addition, Partovi and Razavi also suggested that motivation is influenced by intrinsic phenomena, context, goals, and other factors [44]. The results of the study showed that short video flow would negatively affect students’ motivation to achieve. In short, the negative relationship between short video flow and achievement motivation is supported by both research analysis and the literature.

### 5.3. Short Video Flow Was Positively Related to Short Video Addiction

In this study, the results indicated a positive relationship between short video flow and short video addiction. This also means that H3 is supported. That is, when students experience high levels of flow when watching short videos, they will become more addicted to short videos. This is because when student users experience an enjoyable experience of watching short videos, they will want to re-experience it, so they will want to keep watching them to experience these positive feelings again. This result can be explained by the following literature. As indicated by Kar et al., the use of television and electronic products continues to be prolonged due to the longer duration of pandemic effects, which may lead to behavioral addiction [46]. In addition, Brand et al. noted that according to the I-PACE model, satisfying coping modalities would lead to dysfunction were actively reinforced [18]. Thus, people’s emotional and cognitive responses lead to decisions to act in a particular way [21]. Therefore, when people receive satisfaction from watching short videos, it will reinforce their consumption of short videos, and when there is a problem consuming them, further addiction will ensue. In addition, it is explained from Csikszentmihalyi’s theory of flow that the nature of the flow state is self-enforcing, as people experience positive feelings such as happiness, pleasure, and satisfaction through the consumption of short videos, and are thus motivated to repeat the activity being performed [22]. In short, the positive relationship between short video flow and short video addiction is supported by both research analysis and the literature.

### 5.4. Short Video Addiction Is Positively Related to Serendipity

In this study, the results indicated that short video addiction was positively related to serendipity. This also means that H4 is supported. In other words, the more serious the students felt their addiction to short videos to be, the greater their perceived level of serendipity. This is because short videos are highly entertaining, making it easy for student users to consume them excessively or uncontrollably. When student users become addicted to short videos, it is mostly due to the entertaining nature of the videos’ content. Therefore, student users will indeed experience a sense of instant happiness in the process of watching short videos, which is also in line with the characteristics of short video apps. Such an outcome may also be exacerbated by the characteristics of the pandemic. As indicated by Kar et al., during the pandemic, people spent a great deal of time watching television and their electronic devices because the source of pleasure was limited to indoor activities [46]. That is, people created feelings of happiness by viewing a large amount of information on the Internet. Longstreet and Brooks, on the other hand, suggested that Internet addiction creates a state wherein users feel they need the Internet in order to function in their daily lives [48]. This means obtaining satisfaction by using the Internet continuously. This is also in line with—as suggested by Higgins et al.—the dominant view of happiness or the good life, which is historically viewed as the maximization of pleasure and the minimization of pain [49]. In addition, Gros et al. noted that addiction experts have also observed that addicts tend to confuse serendipity with well-being when associating emotional states with addictive activities [50]. Therefore, addicted people will want to obtain satisfaction and pleasure from the addictive medium. In short, the positive relationship between short video addiction and serendipity is supported by both research analysis and the literature.

### 5.5. Short Video Addiction Is Negatively Related to Achievement Motivation

The findings indicated that short video addiction was negatively related to achievement motivation. This also means that H5 is supported. That is to say, when students feel that they are addicted to watching short videos, their levels of video addition will become more pronounced, and their motivation to achieve will decrease. This is because student users who are in engrossed in a short video cannot control their desire to watch it, and students who pursue entertainment content in short videos will be less likely to be motivated to engage in learning tasks. This result may coincide with the following scholarly views. As inferred from de Barba, motivation can be activated, but it can also be inhibited [51]. Since Internet addiction leads to a gradual decrease in self-determination and eventually to a loss of control over one’s behavior, an important feature of this type of addiction is that it gradually concentrates one’s daily activities in the virtual world [53]. Therefore, addictive substances may interfere with the way people learn, for example, by making students likely to neglect homework [54]. Furthermore, a study by Demir and Kutlu found negative effects of Internet addiction on academic motivation [52]. The findings of Rahayu et al. also confirmed the negative correlation between online game addiction and students’ motivation to learn [36]. A study by Ye et al. also found that short video addiction negatively affected learning motivation. In addition, in the field of motivation-evoking research, it is believed that motivation for achievement needs to be based on achievement goals or achievement incentives [13]; however, short video addiction makes participants want to watch videos repeatedly, which does not lead to academic achievement and may instead lead to academic wastage. Therefore, it also becomes a disincentive for achievement. In short, the negative relationship between short video addiction and achievement motivation is supported by both research analysis and the literature.

### 5.6. Serendipity Is Negatively Related to Achievement Motivation

The results of this study showed that serendipity was negatively associated with achievement motivation. This also means that H6 is supported. That is, the greater the perceptual degree to which students feel serendipity, the lower their achievement motivation will be. This is because one of the characteristics of serendipity is that is allows people to experience happiness in the smallest things, and this will make them easily satisfied. Moreover, in this case, the students’ source of serendipity is short videos, which will also be more detrimental to their drive to develop learning goals. Such results, however, differ from those presented by the previous literature, although San Santoso and Kulathunga pointed out that it is widely believed that happiness can provide a great motivation for someone to achieve higher performance [56]. Therefore, students’ well-being has a positive influence on garnering knowledge, facing new challenges, and sustaining the motivation to learn [57]. A study by Amani Nezhad et al. also found a direct and significant relationship between happiness and achievement motivation among university students [58]. Therefore, Datu et al. argued that serendipity plays an important role in the academic environment [55].

Furthermore, while serendipity has some of the core qualities of happiness, no matter how the idea of serendipity is applied to a given scenario, the characteristics of “easy”, “easy to achieve”, and “easy to satisfy” are, in fact, not separated from it. This concept can be explained by Deci and Ryan’s argument that when individuals feel that their basic psychological needs (i.e., autonomy, competence, and interpersonal relationships) are met, they may be less motivated to achieve greater levels of performance [60]. Thus, while people may be filled with the self-satisfaction of pursuing serendipity, even if there is greater serendipity, this will not result in future benefits with respect to seeking achievement [71]. Maintaining a relaxed, minimally happy work attitude over time may ultimately be detrimental to engagement and performance [24], which is contrary to the notion that the key to achievement motivation is to focus on performing activities well or better than others [59]. As a result, students may not focus on achieving their academic goals when they are chasing serendipity or focusing on the small and beautiful things. In short, the negative relationship between serendipity and achievement motivation is supported by both research analysis and the literature.

## 6. Conclusions and Recommendations

### 6.1. Conclusions and Implications

The I-PACE model is a process model that helps explain Internet usage and outcomes. In this study, an affective–behavioral–outcome model was proposed to explain the antecedents and consequences of short video addiction under the framework of the I-PACE model, and the SEM method was used for model validation. This study shows the following: a. short video flow has a positive relation with serendipity, a negative relation with achievement motivation, and a positive effect on short video addiction; b. short video addiction has a positive effect on serendipity and a negative effect on achievement motivation; and c. serendipity has a negative effect on achievement motivation. This shows that short video addiction, like other Internet addictions, can have a negative impact on students’ ability to learn.

The potential for adverse effects posed by addictive behaviors in the post-epidemic era needs to be carefully considered from the perspective of developing public health strategies. Therefore, there is a need to decrease the student community’s dependence on short videos in order to help prevent an increase in addictive behavior. The results of this study may be of some use for mental health organizations and educational institutions that are designing programs to assist in the prevention of short video addiction among youth during the COVID-19 pandemic. This is the practical contribution of this study. In addition, short video addiction is an emerging research topic and there is still much to be explored in the empirical research on this topic. Further research is still needed to help explain the association between short video addiction and learning. The results of this study may help to expand the understanding of short video addiction research, which is the theoretical contribution of this study.

From the traditional viewpoint of positive psychology, positive experiences lead to better outcomes for people. However, this is not necessarily the case in this study. This is one of the important research findings and theoretical contributions of this study. In addition, serendipity is a concept pursued by young people in East Asia. Although serendipity is often mentioned in the lives of people in Asia and seen in many advertisements, it is still a very new term in the field of social science. This is the first international study to confirm the impact of serendipity on students. Although this particular concept brings a subjective positive feeling to the person concerned and allows him/her to experience a very short period of happiness, it often has a negative connotation in the objective thoughts of third parties, because in the eyes of third parties, after experiencing serendipity, people often tend to be satisfied with the status quo or pursue serendipity excessively. In the present study, it was also confirmed that too high a level of serendipity would inhibit achievement motivation.

In addition, as one of the world’s largest economies and a country with global influence, people from across the globe are very curious about the current situation in China. However, Chinese culture is very different from that of Western countries. Therefore, a growing number of scholars are now devoting themselves to this area of research in order to better explain the Chinese context. While contemporary international Chinese Studies have been extended to the field of education and psychology, this study also analyzes the impact of research variables with east Asian elements (serendipity) on the Chinese student population, which will help the international academic community to better understand the situation in China. This is also the theoretical contribution of this study.

### 6.2. Recommendations

While short videos are entertaining and relaxing in a simple way, their immersive nature should not be underestimated. Although short video addiction provides social entertainment to users, which is fine for most users, it will be a serious problem for addicted users who cannot control themselves. From the results of the study, it is clear that short video flow is an important antecedent of short video addiction, but allowing users to experience flow is a characteristic of short video media. Therefore, watchers of short videos should change their habits to effectively avoid becoming too immersed.

In addition, this study also confirmed that serendipity negatively affects students’ motivation to achieve. Therefore, it is also suggested that parents or teachers should inform students that they should not pursue serendipity excessively and should avoid being content with the status quo, as these states may lead to weak motivation to achieve goals.

### 6.3. Limitations and Future Study

The results of the current study should be interpreted with caution given the following limitations: the respondents were recruited using convenience sampling, which may reduce the generalizability and replicability of the findings, and due to the cross-sectional design of the current study, significantly associated findings do not support any causal relationships [72]. In addition, because of the cross-sectional design of this study, it is not possible to exclude the possibility that the participants in this study had already had short video addiction before the COVID-19 pandemic.

Second, all the data collected in this study were obtained through self-reporting, which heavily relies on participants’ clear understanding of the issues and honest reporting and may be affected by response bias. Therefore, in the future, evaluation and validation can be conducted with the help of other objective data to examine whether there is a discrepancy between objective data and the self-reported impacts of short video addiction. At the same time, the various types of Internet addiction can still be explored to help us understand the impact of these behavioral addictions on students’ learning, life, and mental health.

Third, in this study, flow was used as a single dimensional construct. However, the flow experience can also be considered as a continuum with multidimensional characteristics. Therefore, in follow-up studies, the multidimensional flow experience can be used as a core variable to explore the causes of Internet addiction, including Short video addiction, which will help to understand the causes of behavioral addiction.

Fourth, serendipity is a new research topic, and the existing literature on this topic mostly consists of the personal opinions of scholars. There are few empirical studies on serendipity; thus, there is still much to explore on this topic. Therefore, it is suggested that in the future, interviews could be conducted with student groups regarding their definitions and perceived effects of serendipity, as well as among teachers or parents to determine their perceptions of serendipity.

Finally, Chinese studies is an important contemporary international field of integrated studies, and exploring China as a contextual background will help to expand the international academic community’s understanding of the influence of Chinese culture. Therefore, it is suggested that further research continues to define and explore more China-specific research variables, concepts, and theories to understand their impacts.

## Figures and Tables

**Table 1:** First-order Confirmatory Factor Analysis.

Index	Threshold	Short Video Flow	Short Video Addiction	Serendipity	Achievement Motivation
χ^2^	---	6.7	23.8	21.4	10.4
*df*	---	5	5	5	5
χ^2^/*df*	<5	1.34	4.76	4.28	2.08
RMSEA	<0.10	0.02	0.06	0.06	0.03
GFI	>0.80	0.99	0.99	0.99	0.99
AGFI	>0.80	0.99	0.97	0.97	0.99
FL	>0.50	0.72~0.84	0.64~0.71	0.65~0.92	0.57~0.90
*t*	>3	25.75~36.76	24.40~29.34	28.52~34.11	23.25~31.86

Note: χ^2^ = Chi-Square, *df* = degrees of freedom, χ^2^/*df* = Chi-Square/degrees of freedom, RMSEA = root-mean-square error of approximation, GFI = goodness of fit index, AGFI = adjusted goodness of fit index, and FL = factor loadings.

**Table 2:** Reliability and Validity Analysis.

Construct	M	SD	α	CR	AVE	FL
	---	---	>0.70	>0.70	>0.50	>0.50
Short video flow	2.71	0.91	0.87	0.87	0.58	0.76
Short video addiction	2.15	0.76	0.81	0.81	0.50	0.68
Serendipity	3.14	0.86	0.92	0.92	0.70	0.81
Achievement motivation	3.56	0.72	0.85	0.86	0.56	0.73

Note: M = mean, SD = standard deviation, α = Cronbach’s α, CR = composite reliability, AVE = average variance extracted, and FL = factor loadings.

**Table 3:** Discriminant Construct Validity.

Construct	1	2	3	4
1. Short video flow	(0.76)			
2. Short video addiction	0.608	(0.75)		
3. Serendipity	0.474	0.415	(0.84)	
4. Achievement motivation	−0.327	−0.287	−0.279	(74)

Note: The value on the diagonal line is the square root value of AVE, while the other values are the related coefficient values.

## Data Availability

The original contributions presented in the study are included in the article; further inquiries can be directed to the corresponding author.
